# Association of *IL10RA*, *IL10RB*, and *IL22RA* Polymorphisms/Haplotypes with Susceptibility to and Clinical Manifestations of SLE

**DOI:** 10.3390/ijms241411292

**Published:** 2023-07-10

**Authors:** Milka Grk, Rada Miskovic, Ivica Jeremic, Milica Basaric, Marija Dusanovic Pjevic, Biljana Jekic, Danijela Miljanovic, Ivana Lazarevic, Aleksa Despotovic, Andja Cirkovic, Ana Banko

**Affiliations:** 1 Institute of Human Genetics, Faculty of Medicine, University of Belgrade, 11000 Belgrade, Serbia; milka.grk@med.bg.ac.rs (M.G.);; 2 Clinic of Allergy and Immunology, University Clinical Center of Serbia, 11000 Belgrade, Serbia; 3 Internal Medicine Department, Faculty of Medicine, University of Belgrade, 11000 Belgrade, Serbia; 4 Institute of Rheumatology, Faculty of Medicine, University of Belgrade, 11000 Belgrade, Serbia; 5 Institute of Microbiology and Immunology, Faculty of Medicine, University of Belgrade, 11000 Belgrade, Serbia; 6 Institute for Medical Statistics and Informatics, Faculty of Medicine, University of Belgrade, 11000 Belgrade, Serbia

**Keywords:** systemic lupus erythematosus, IL10RA, IL10RB, IL22RA, polymorphism, Hashimoto thyroiditis

## Abstract

Systemic lupus erythematosus (SLE) is characterized by an imbalance between proinflammatory and anti-inflammatory mediators. Single-nucleotide polymorphisms (SNPs) in genes coding *IL10RA*, *IL10RB*, and *IL22RA* could affect their expression or function and disrupt immune homeostasis. We aimed to analyze the associations of *IL10RA*, *IL10RB*, and *IL22RA* polymorphisms/haplotypes with patients’ susceptibility to and clinical manifestations of SLE. Our study included 103 SLE patients and 99 healthy controls. The genotypes of the selected polymorphisms within *IL10RA* (rs10892202, rs4252270, rs3135932, rs2228055, rs2229113, and rs9610), *IL10RB* (rs999788, rs2834167, and rs1058867), and *IL22RA* (rs3795299 and rs16829204) genes were determined by TaqMan^®^ Assays. *IL10RB* rs1058867 G allele carriers were significantly more frequent among the controls than among the SLE patients (76.8% vs. 61.2%; *p* = 0.017, OR = 0.477, 95% CI: 0.258–0.879). The *IL10RB* CAA haplotype was more frequent among the SLE patients than in the control group (42.7% vs. 30.7%; *p* = 0.027). The *IL22RA* rs3795299 C allele and rs16829204 CC genotype were associated with Hashimoto thyroiditis in the SLE patients (n = 103; *p* = 0.002 and *p* = 0.026, respectively), and in all the included participants (n = 202, *p* < 0.000 and *p* = 0.007, respectively), and the *IL22RA* CC haplotype was more frequent in the SLE patients with Hashimoto thyroiditis (*p* = 0.047) and in the overall participants with Hashimoto thyroiditis (n = 32, *p* = 0.004). The *IL10RA*, *IL10RB*, and *IL22RA* polymorphisms/haplotypes could be associated with SLE susceptibility and various clinical manifestations, and the *IL22RA* CC haplotype could be associated with Hashimoto thyroiditis.

## 1. Introduction

Systemic lupus erythematosus (SLE) is a chronic, multisystemic autoimmune disease with heterogeneous clinical manifestations associated with autoantibody production, immune complex formation and deposition, and other immune processes [1,2]. There is a significantly higher prevalence in woman, and the female to male ratio goes up to 15:1 for women at reproductive age [3]. Its etiology is not fully elucidated, but it is well established that this multifactorial disease results from a complex interaction between genetic and environmental factors [2,4]. Genetic factors could predetermine subtle changes in immune system cell signaling and disrupt the balance between pro-inflammatory and anti-inflammatory pathways [4]. Interleukin 10 (IL10) is a potent anti-inflammatory cytokine which plays an important role in immunoregulation. It has also been shown that IL10 promotes B lymphocyte differentiation, proliferation, and class switching [5].

IL10 exerts its effect via the IL10 receptor (IL10R). This receptor is a heterotetramer composed of two IL10R A subunits (IL10RA) and two IL10R B subunits (IL10RB). Both molecules are members of the class II cytokine receptor family (CRF2) [6,7]. After IL10 binds to IL10RA, this complex changes conformation and allows for the binding of the IL10rb subunit of the receptor [6]. As a result, the JAK1-TIK2-STAT3 signaling cascade is triggered [8,9]. Although STAT3 is the main downstream transcription factor, IL10 also activates STAT1 and STAT5 [10]. Except for the suppression of proinflammatory cytokine production, this pathway leads to decreased macrophage activity, the inhibition of monocyte differentiation to dendritic cells, and the downregulation of costimulatory molecules on antigen-presenting cells [5,9]. Meanwhile, B lymphocyte stimulation by IL-10 results in pro-inflammatory behavior [11,12,13].

While the IL10RA subunit is present mainly in leukocytes and is specific for IL10RA, the IL10R B subunit is ubiquitous and participates in the building of receptor complexes for IL22, IL26, IL28a, IL28b, and IL29 [8,14]. Single-nucleotide polymorphisms (SNPs) in gene-coding IL10R subunits could affect their interaction as well as signaling through this pathway and could disrupt immune homeostasis. As cytokines play a crucial role in the regulation of our immune system, we aimed to assess the associations between selected polymorphisms in the IL10RA, IL10RB, and IL22RA genes with SLE susceptibility and its clinical manifestations.

## 2. Results

### 2.1. Demographic and Clinical Data

The demographic data and clinical manifestations in the control group and the SLE patients are shown in Table 1 and Table 2. The median disease duration in our SLE patients was five years (0.0–42.0). Among the SLE patients, 41 (39.8%) had high disease activity with a clinical SLEDAI score above 4. Corticosteroid therapy was used by 90 (87.4%) of the patients with a median daily prednisone dosage of 12.5 mg (0–80), 86 (83.5%) patients used hydroxychloroquine, 30 (29.1%) had had immunosuppressants (AZA, MTX, MMF) during treatment, and pulse cyclophosphamide and/or glucocorticoids therapy was administered in 13 (12.7%) patients. Secondary Sjogren syndrome was present in 39 (37.9%) of the SLE patients while secondary APS was observed in 9 (8.7%) of the SLE patients. The damage accumulated since the onset of lupus was assessed using the SLICC/ACR damage index, and in our patients, it ranged between 0.0 and 6.0 with a median value of 0.0.

### 2.2. Genotype/Haplotype Analysis and SLE Susceptibility

#### 2.2.1. Genotype Analysis

The frequencies of the genotypes/alleles for all of the investigated polymorphisms within the *IL10RA*, *IL10RB*, and *IL22RA* genes are presented in Table 3. The genotype distributions of the studied polymorphisms did not deviate from the Hardy–Weinberg equilibrium in the SLE patients and the control group. *IL10RB* rs1058867 G allele carriers were significantly more frequent among the controls than among the SLE patients (76.8% vs. 61.2%; *p* = 0.038, OR = 0.477, 95% CI: 0.258–0.879). This result was significant after using a logistic regression analysis adjusted for age and gender.

#### 2.2.2. Haplotype Analysis

Additionally, a haplotype analysis of the *IL10RA* (rs10892202, rs4252270, rs3135932, rs2228055, rs2229113, and rs9610), *IL10RB* (rs999788, rs2834167, and rs1058867) and *IL22RA* (rs3795299 and rs16829204) genes was performed.

##### *IL10RA* Gene Haplotypes

Haplotype blocks among all of the analyzed IL10RA polymorphisms were observed when all subjects of the study were included (n = 202), while in the SLE patients, haplotype blocks were observed between rs10892202 and rs4252270 (D’ = 1, r^2^ = 1) and between rs2229113 and rs9610 polymorphisms (D’ = 0.98, r^2^ = 0.42). In the control group, a haplotype block was present between rs10892202 and rs4252270 polymorphisms (D’ = 1, r^2^ = 1). In the SLE patients, GCAAGA, GCAAAG, GCGAAG, CTAAGG, GCAGGG, and GCAAGG haplotypes were present in 47.1%, 19%, 16.4%, 7.3%, 5.3%, and 4.3%, respectively, and in the control group, the same haplotypes had frequencies of 48.4%, 17.2%, 10.6%, 9.1%, 7.6%, and 6.6%, respectively. There was no significant difference in the haplotype frequencies between these two groups.

##### *IL10RB* Gene Haplotypes

**Figure 1:**
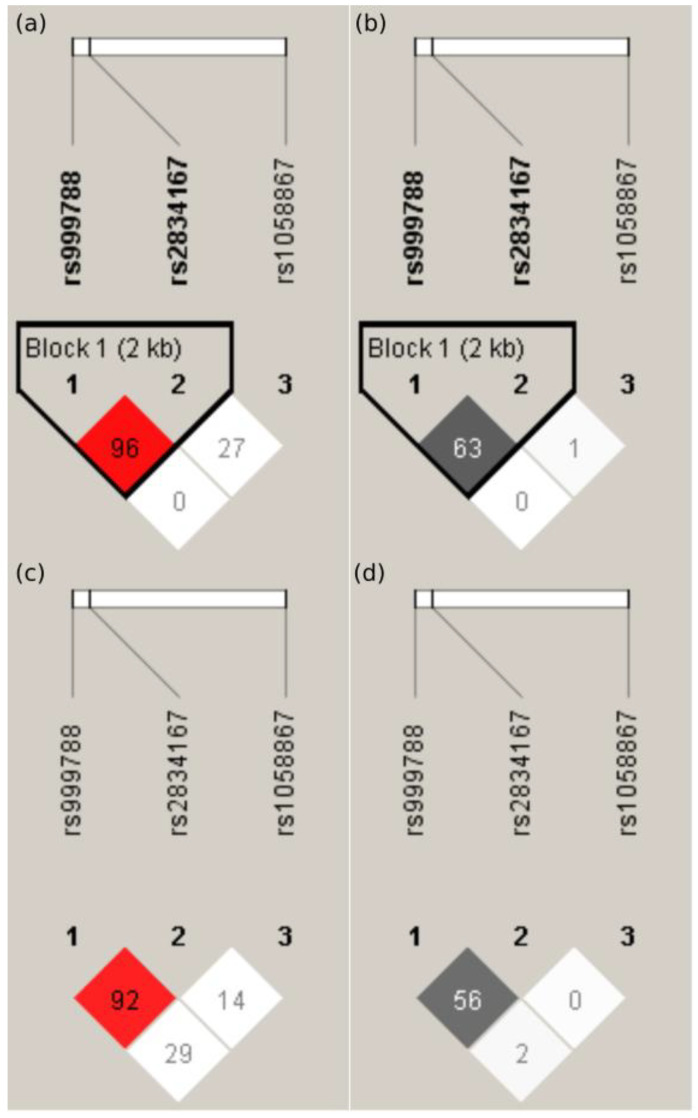
Schematic representation of LD with D’ (**a**,**c**) and r^2^ (**b**,**d**) values among the analyzed *IL10RB* gene polymorphisms (rs999788, rs2834167, and rs1058867) in SLE patients (**a**,**b**) and in the control group (**c**,**d**).

The D’ and r^2^ values in the SLE patients and the control group are shown in Figure 1. Haplotype blocks were present between rs999788 and rs2834167 polymorphisms in the SLE patients. The CAA, CAG, TGA, TGG, CGA, and CGG haplotypes were harbored by 42.7%, 30.1%, 12.2%, 7.2%, 6.3%, and 1.1% of the SLE patients, respectively, while the CAG, CAA, TGA, TGG, CGG, CGA, and TAA haplotypes (39.4%, 30.7%, 12.5%, 7.6%, 4.5%, 4.2%, and 1.1%, respectively) were present in the control group. The *IL10RB* CAA haplotype was more frequent among the SLE patients than in the control group (42.7% vs. 30.7%; *p* = 0.027).

##### *IL22RA* Haplotypes

Haplotype blocks were not observed between the analyzed *IL22RA* polymorphisms (rs16829204 and rs3795299; D’ = 0.8, r^2^ = 0.07). The GC, CC, GT, and CT haplotypes were present in the SLE patients with a frequency of 49.3%, 32.2%, 16.2%, and 2.2%, respectively, while in the control group, the frequencies of the GC, CC, and GT haplotypes were 48%, 39.4%, and 12.6%, respectively. The haplotype frequencies of the patients and the control group did not differ significantly.

### 2.3. Genotype/Haplotype Analysis and Clinical Manifestation in SLE Patients

The association of the analyzed polymorphisms and the clinical manifestations in the SLE patients are shown in Table 4.

Additionally, we included both the controls and SLE patients (n = 202) and analyzed the association between rs16829204, rs3795299, and Hashimoto thyroiditis. We confirmed that among the participants with the rs16829204 CC genotype, Hashimoto thyroiditis was more frequent than in those with T allele carriers (20.4% vs. 5.1%; *p* = 0.007, OR = 4.79, 95% CI: 1.399–16.407), and that among the rs3795299 C allele carriers, Hashimoto thyroiditis was more frequent than in those with GG genotype carriers (23.5% vs. 4.9%; *p* < 0.000, OR = 6, 95% CI: 2.016–17.853). This result remains significant after a multivariate logistic regression. An additional analysis showed that the *IL22RA* CC haplotype was more frequent (48.4% vs. 33%; *p* = 0.004) in the participants with Hashimoto thyroiditis (n = 32).

The haplotype analysis showed that the SLE patients without Sjogren syndrome had the *IL10RA* GCGAAG haplotype significantly more frequently than the patients with Sjogren syndrome (21.9% vs. 7%, *p* = 0.007). The patients without Hashimoto thyroiditis had *IL10RA* GCAAGA and *IL22RA* GT haplotypes significantly more frequently than the patients with Hashimoto thyroiditis (50.6% vs. 29.4%; *p* = 0.024 and 19.6% vs. 0%; 0.016, respectively). At the same time, the *IL22RA* CC haplotype was more frequent in the SLE patients with Hashimoto thyroiditis (44.1% vs. 29.6%; *p* = 0.047). The patients without a malar rash had the *IL10RA* CTAAGG, *IL10RB* TGG, and *IL22RA* GC haplotypes more frequently than the patients with a malar rash (*p* = 0.009, *p* = 0.031, *p* = 0.049, respectively).

The *IL10RA* GCAAGA haplotype was more frequent in patients with than in patients without alopecia (56.2% vs. 41.3%; *p* = 0.036). The *IL10RA* GCAGGG haplotype was more frequent in patients with pathological urine sediment than in patients with normal findings (16.7% vs. 4.3%; *p* = 0.025). The *IL10RB* CAA haplotype was more frequent in patients with oral ulcerations than in patients without this classification criteria (54.3% vs 38.9%; *p* = 0.038). The *IL10RB* CGA haplotype was more frequent in patients with thrombocytopenia and lupus nephritis than in patients without these manifestations (12.5% vs. 4.3%; *p* = 0.045 and 13% vs. 4.2%; *p* = 0.030, respectively), while this haplotype was less frequent in patients with than in patients without arthritis (20% vs. 4.8%; *p* = 0.008).

## 3. Discussion

SLE is a multifactorial disease with a genetic basis and a pathogenesis that has not fully been elucidated yet [4]. Numerous gene variants are associated with SLE susceptibility [15]. It is known that SLE is characterized by an imbalance between the production of pro-inflammatory and anti-inflammatory cytokines [1,2]. IL10 was singled out as one of the essential immunomodulatory cytokines [16]. It has been known for years that polymorphisms located in the *IL10* gene promoter (rs1800896, rs1800871, and rs1800872) affect gene expression [17,18]. Previous studies have shown that the IL10 levels are higher in the blood of patients with SLE than in healthy individuals. The IL10 levels correlate well with the disease activity estimated through the SLEDAI score [17,19,20,21]. Meanwhile, only a few studies have examined the influence of polymorphisms in gene-coding IL10 receptors on patients’ susceptibility to and clinical course of SLE [14,22].

According to our research, only one study has analyzed the association of rs3135932 and rs2229113 *IL10RA* polymorphisms and SLE, and one study has analyzed the rs2834167 *IL10RB* polymorphism and SLE [14,22]. Hermann et al. showed that both the rs3135932 (S138G) and rs2229113 (G330R) polymorphisms are loss-of-function variants and lead to reduced IL10RA signal transduction. In this study, rs2229113 was associated with RA and SLE susceptibility, while the influence of rs3135932 was not significant [22].

We included six *IL10RA* polymorphisms: rs10892202, rs4252270, rs3135932, rs2228055, rs2229113, and rs9610. These polymorphisms and the *IL10RA* haplotypes did not show an association with the patients’ SLE susceptibility. At the same time, we investigated the association of *IL10RA* polymorphisms with clinical manifestations in SLE patients. Skin manifestations were among the most common in our patients. The malar rash was associated with rs10892202, rs4252270, and their GC haplotype. The *IL10RA* rs2229113 polymorphism GG genotype was associated with photosensitivity. Although rs10892202 was initially associated with discoid manifestations, a combined genotype and allele analysis did not confirm its influence. Arthritis was associated with the rs9610 G allele. According to Peng et al., in the Chinese population, the rs9610 A allele is associated with RA susceptibility [23]. These two results are difficult to compare due to the different pathogenesis of erosive arthritis in RA and non-erosive arthritis in SLE, as well as the difference in the genetic basis of the two populations, which can be seen through the distribution of the rs9610 allele (MAF(G) = 0.45 in East Asia; MAF(A) = 0.45 in Europe) [24]. Patients with hemolytic anemia carry the rs10892202 C allele and rs4252270 T allele more often than controls. Due to the small number of patients with hemolytic anemia, this result should be confirmed on a larger number of patients. These alleles had a protective effect on the early onset of IBD [25].

Peng et al. associated the rs2834167 allele A with SLE susceptibility [14]. In our study, this polymorphism was not significant in this manner, and this could be the result of the different genetic backgrounds of the Chinese Han and European populations. According to the 1000-genome study, allele A has significantly different frequencies in East Asian and European populations (0.44 vs. 0.73) [26]. On the other hand, the *IL10RB* CAA haplotype (rs999788, rs2834167, and rs1058867) was more frequent in the SLE patients than in the control group. In our study, rs1058867 allele A was significantly associated with SLE, and this polymorphism could carry the significance of the entire haplotype. Still, we must consider the possibility that the remaining two polymorphisms have a subtle influence that could not be seen due to the small number of study participants. Our study is the first to assess the *IL10RB* polymorphism’s influence on the clinical manifestation of this disease. In our study, the rs2834167 AA genotype was associated with malar rashes, the same as the rs999788 CC genotype. Hikami et al. associated the rs999788 CC genotype and rs2834167 AA genotype with systemic sclerosis. These two polymorphisms showed strong LD in Hikami et al.’s study and in our study [27]. Allele A (47lys) showed an association with a lower *IL10RB* expression in Frodshman et al.’s research, possibly due to strong LD with rs999788, located in the NF-κβ binding site [27,28]. This binding site may be compromised in presence of C allele [27]. Polymorphism rs1058867 is located in the 3′ UTR region and according to our study, it is not strongly linked with rs999788 and rs2834167. Polymorphisms in the 3′ UTR region could affect protein expression through changes in mRNA stability and its interaction with regulatory proteins, and miRNA [29]. After a variant in the non-coding region affects the receptor expression or transduction, changes in the production of IL10, as seen in SLE, could be a response to receptor availability or malfunction. Consequently, the control of pro-inflammatory cytokines by IL10 would be disrupted. Such an imbalance could result in an increased tendency towards an altered immune response and the development of autoimmune diseases such as SLE.

An additional haplotype analysis revealed an association of the *IL10RA* and *IL10RB* haplotypes with the clinical course of disease. As a multisystemic disease, SLE is characterized by heterogenous manifestations frequently followed by the development of secondary autoimmune entities, such as secondary antiphospholipid disease, Sjogren syndrome, and thyroiditis [30]. Our study showed that the *IL10RA* GCGAAG haplotype was more frequent in the SLE patients without secondary Sjogren syndrome, and the GCAAGA haplotype was more frequent in the SLE patients without secondary Hashimoto thyroiditis. These haplotypes could be protective for the development of secondary entities in SLE patients. Interestingly, some of haplotypes, such as the *IL10RB* CGA haplotype, acted protectively toward arthritis occurrence, and at the same time, this haplotype was more frequent in the patients with thrombocytopenia and nephritis.

Our study showed that the *IL22RA* rs3795299 C allele and rs16829204 CC genotype are associated with Hashimoto thyroiditis in SLE patients and in all included participants, and we confirmed these results in our haplotype analysis. The *IL22RA* gene rs3795299 (c.1552C > G, p.Arg518Gly) and rs16829204 (c.613G > A p.Val205ILe) polymorphisms are both missense variants, and this can explain their effect on *IL22RA* function [26,31,32]. This is the first study that analyzed the effect of *IL22RA* polymorphisms on the development and clinical manifestation of autoimmune diseases. Niu et al. showed that the rs3795299 G allele protects preeclampsia development in Chinese Han women [32]. Dinarte et al. found no significant association between rs16829204 and chronic rhinosinusitis [31].

The limitation of our study that needs to be acknowledged was the relatively small number of patients, which reflects the number of inhabitants of our country. We would like to emphasize that this is the first study that has examined the variants within the *IL10RA*, *IL10RB*, and *IL22RA* genes and their association with SLE susceptibility and the disease’s manifestations. We would like for this study to be the basis for further research that includes a larger number of patients and contributes to the elucidation of the enigmatic role of anti-inflammatory pathways in the development of autoimmune diseases.

## 4. Materials and Methods

### 4.1. Patients and Controls

All 103 SLE patients in the study fulfilled the American College of Rheumatology (ACR) 1997 classification criteria for SLE [33]. All patients were diagnosed, treated, and prospectively followed up with at the Clinic for Allergology and Immunology University Clinical Center of Serbia and the Institute of Rheumatology, Belgrade between June 2020 and November 2021. Disease activity was assessed using the SLEDAI 2K scale, and Physician Global Assessment (PGA) [34,35]. Demographic, clinical, and laboratory data were collected from each patient at the time of their enrollment in the study. We collected the following data on the presence of antibodies in each patient: antinuclear (ANA), anti-dsDNA antibodies, anti-Sm, anti-SSA, anti-cardiolipin antibodies (aCL), anti-beta2-glycoprotein I (anti-β2-GPI) IgG and IgM, anticardiolipin IgG/IgM, anti-β2-GPI IgG/IgM, anti-SSA and anti-Sm, and rheumatoid factor (RF). ANA titer above 1:80 was considered positive. Serum concentrations of C3, C4, IgG, IgM, and IgA were recorded. The exclusion criteria for both study cohorts were those who were under 18, who were inable to cooperate, and who had malignancies. Patients with significant comorbidities (severe cardiac, pulmonary, and psychiatric diseases or dementia) were excluded from the study. All patients gave written informed consent to participate in the study. The study was carried out according to the Declaration of Helsinki and approved by the Ethical Board of the Faculty of Medicine, University of Belgrade (No. 1550/IX-14). The control group included 99 participants who did not suffer from any autoimmune rheumatology disease with negative family history of these diseases.

### 4.2. Genotyping

Molecular-genetic analyses were performed at the Institute of Human Genetics, Faculty of Medicine, University of Belgrade. Total genomic DNA was extracted from peripheral blood leukocytes using the standard salting out method [36]. DNA concentration and quality were checked photospectrometrically. Genotypes within *IL10RA* (rs10892202, rs4252270, rs3135932, rs2228055, rs2229113, and rs9610), *IL10RB* (rs999788, rs2834167, and rs1058867) and *IL22RA* (rs3795299 and rs16829204) were determined using TaqMan^®^ SNP Genotyping Assays (Applied Biosystems, Foster City, CA, USA) and Applied Biosystems’ 7500 Real-Time PCR System (Applied Biosystems).

### 4.3. Data Analysis

Depending on the data distribution, numerical data are described with the arithmetic mean and standard deviation or median and range. Normal distribution was evaluated by mathematical (Kolmogorov–Smirnov and Shapiro–Wilk test, skewness and kurtosis, and coefficient of variation) and graphical methods (histogram, box plot, and Q-Q diagram). Categorical variables were presented as an absolute and relative numbers in the form of n (%). Student’s *t*-test for two independent samples or Mann–Whitney U test were used to compare numerical data between study cohorts, depending of the data distribution. The Chi-square test was used to test the difference in the distribution of categories of two independent samples.

In order to evaluate all possible factors influencing one’s probability of having systematic lupus erythematosus, univariate logistic regression analysis was performed first, with multivariate analysis thereafter with significant variables from the previous analysis in the model. VIF multicollinearity testing method and correlation coefficients were used. All variables with VIF > 5 were eliminated from the multivariate model. moreover, according to the covariance matrix and correlation coefficients, one of two correlated variables with a lower *p*-value in univariate logistic regression was eliminated. Forward (Wald) regression method was applied, and only the last step was presented in the results. Odds ratio (OR), 95% confidence interval of odds ratio (95% CI OR), and *p*-value were reported. The sample size was calculated by G Power 3.1.9.2 for the major outcomes. According to previously known data about the *IL10RB* rs1058867 polymorphism’s allele distribution given by 1000 Genomes Project Consortium for a medium effect size of 0.3, α of 0.05, power of 80%, and df = 1, we obtained a total sample size per group of 88 [24].

Haplotype analysis was performed using HaploView 4.2 software [37]. Haplotype blocks were evaluated using the “Confidence Intervals LD” method.

## 5. Conclusions

Our study showed that the polymorphisms/haplotypes in the genes coding IL10R could contribute to SLE development. Additionally, the result of this study indicates the possibility that specific genotypes/haplotypes can direct the course of the disease towards specific clinical manifestations. Such knowledge could help in the development of personalized medicine, providing a valuable tool for monitoring high-risk patients with a goal of the early detection of the first signs of the development of severe disease manifestations.

## Figures and Tables

**Table 1:** Baseline characteristics of the study cohorts.

Characteristic	Group		*p* *
	SLE n = 103	Control n = 99	
Age (years), mean ± sd	45.43 ± 12.90	55.43 ± 13.62	<0.001 £
Female gender, n (%)	94 (91.3)	87 (87.9)	0.431 §
BMI, mean ± sd	25.93 ± 5.25	27.18 ± 4.54	0.074 £
Smokers, n (%)	60 (60.0)	39 (39.4)	0.004 §
Smoking duration (years), med (min–max)	16.0 (0.5–40.0)	0.0 (0.0–45.0)	<0.001 ¥
Hashimoto thyroiditis, n (%)	17 (16.7)	15 (15.2)	0.769 §
HTA, n (%)	44 (42.7)	47 (47.5)	0.497 §
DM, n (%)	7 (6.8)	11 (11.1)	0.282 §
Cardiovascular events (CVI, TIA, AIM, AP), n (%)	9 (8.7)	17 (17.2)	0.074 §

* For the level of significance of 0.05, according to Student *t*-test £, Chi-square test §, and Mann–Whitney U test ¥. BMI—body mass index; HTA—arterial hypertension; DM—diabetes mellitus; CVI—cerebrovascular insult; TIA—transient ischemic attack; AIM—acute myocardial infarction; AP—angina pectoris.

**Table 2:** Clinical and laboratory data of the SLE patients.

ACR 1997 Criteria, n (%)	
Malar rash	60 (58.3)
Photosensitivity	72 (69.9)
Discoid rash	41 (39.8)
Oral ulcerations	26 (25.2)
Nonerosive arthritis	92 (89.3)
Serositis (pleuritis or pericarditis)	15 (14.6)
Renal disorder	25 (24.3)
Neurologic disorder	4 (3.9)
Hematologic disorder	73 (70.9)
ANA	102 (99)
Immunologic disorders	70 (68)
Number of positive criteria, med (min–max)	6.0 (4.0–11.0)
Immunological parameters	
ANA, titer values, med (min–max)	640.0 (40.0–640.0)
The presence of Anti-dsDNA, n (%)	30 (29.1)
Anti-dsDNA, titer values, med (min–max)	0.0 (0.0–640.0)
Anti-SSA, n/N (%)	43/99 (43.4)
Anti-SSA (U/mL), med (min–max)	4.9 (0.0–360.0)
Anti-Sm, n/N (%)	22/101 (21.8)
Anti-Sm, med (min–max)	0.6 (0.0–300.0)
aCL-IgM, n/N (%)	14/100 (14.0)
aCL-IgM (MPL), med (min–max)	1.3 (0.0–170.0)
aCL-IgG, n/N (%)	14/100 (14.0)
aCL-IgG (GPL), med (min–max)	3.45 (0.0–251.8)
Anti-β2-GPI IgG, n (%)	7 (7.4)
Anti-β2-GPI IgM, n (%)	7 (7.4)
RF, n/N (%)	19/94 (20.2)
RF (IU/mL), med (min–max)	20.0 (0.0–561.0)
C3 (g/L), (mean ± sd)	0.81 ± 0.27
C4 (g/L), med (min-max)	0.12 (0.02–0.87)
Low C3 or/and C4, n (%)	59 (57.3)
Total IgG (g/L), (mean ± sd)	13.91 ± 5.20
FACIT score	35.51 ± 12.05
Laboratory assessment, med (min–max)	
ESR (mm/h)	22.0 (4.0–125.0)
CRP (mg/L)	2.8 (0.2–87.5)
NLR	2.1 (0.0–15.3)
Disease activity assessment, med (min–max)	
SLEDAI 2K	5.0 (0.0–29.0)
Serologic SLEDAI 2K	2.0 (0.0–5.0)
Clinical SLEDAI 2K	3.0 (0.0–25.0)
PGA	28.0 (0.0–78.0)

ESR—erythrocyte sedimentation rate; CRP—C reactive protein; NLR—neutrophil to lymphocyte ratio; SLEDAI 2K—Systemic Lupus Erythematosus Disease Activity Index 2000; PGA—Physician Global Assessment.

**Table 3:** Associations between the *IL10RA*, *IL10RB*, and *IL22RA* gene polymorphisms and SLE.

Polymorphisms	Control Group n (%)	SLE Patients n (%)	*p*-Values *
*IL10RA* rs10892202			
GG	83 (83.8)	89 (86.4)	0.779
GC	14 (14.1)	13 (12.6)	
CC	2 (2)	1 (1)	
G	180 (90.9)	191 (92.7)	0.587
C	18 (9.1)	15 (7.3)	
*IL10RA* rs4252270			
CC	83 (83.8)	89 (86.4)	0.779
CT	14 (14.1)	13 (12.6)	
TT	2 (2)	1 (1)	
C	180 (90.9)	191 (92.7)	0.587
T	18 (9.1)	15 (7.3)	
*IL10RA* rs3135932			
AA	77 (77.8)	72 (69.9)	0.100
AG	22 (22.2)	27 (26.2)	
GG	0 (0)	4 (3.9)	
A	176 (88.9)	171 (83)	0.115
G	22 (11.1)	35 (17)	
*IL10RA* rs2228055			
AA	84 (84.8)	92 (89.3)	0.343
AG	15 (15.2)	11 (10.7)	
GG	0 (0)	0 (0)	
A	183 (92.4)	195 (94.7)	0.420
G	15 (7.6)	11 (5.3)	
*IL10RA* rs2229113			
GG	49 (49.5)	44 (42.7)	0.182
AG	44 (44.4)	45 (43.7)	
AA	6 (6.1)	14 (13.6)	
G	142 (71.7)	133 (64.6)	0.136
A	56 (28.3)	73 (35.4)	
*IL10RA* rs9610			
AA	24 (24.2)	25 (24.3)	0.810
AG	49 (49.5)	47 (45.6)	
GG	26 (26.3)	31 (30.1)	
A	97 (49)	97 (47.1)	0.765
G	101 (51)	109 (52.9)	
*IL10RB* rs999788			
CC	60 (60.6)	66 (64.1)	0.787
CT	36 (36.4)	33 (32)	
TT	3 (3)	4 (3.9)	
C	156 (78.8)	165 (80.1)	0.806
T	42 (21.2)	41 (19.9)	
*IL10RB* rs2834167			
AA	47 (47.5)	54 (52.4)	0.714
AG	47 (47.5)	43 (41.7)	
GG	5 (5.1)	6 (5.8)	
A	141 (71.2)	151 (73.3)	0.658
G	57 (28.8)	55 (26.7)	
*IL10RB* rs1058867			
AA	23 (23.2)	40 (38.8)	0.038
AG	50 (50.5)	46 (44.7)	
GG	26 (26.3)	17 (16.5)	
A	96 (48.4)	126 (61.2)	0.012
G	102 (51.5)	80 (38.8)	
*IL22RA* rs16829204			
CC	76 (76.8)	67 (65)	0.169
CT	21 (21.2)	34 (33)	
TT	2 (2)	2 (1.9)	
C	173 (87.4)	168 (81.6)	0.131
T	25 (12.6)	38 (18.4)	
*IL22RA* rs3795299			
CC	17 (17.2)	12 (11.7)	0.517
GC	44 (44.4)	47 (45.6)	
GG	38 (38.4)	44 (42.7)	
C	78 (39.4)	71 (34.5)	0.353
G	120 (60.6)	135 (65.5)	

* For the level of significance of 0.05, according to Chi-square test or Fisher’s exact test.

**Table 4:** Polymorphism-Associated Clinical Manifestations in SLE Patients.

Manifestation	Predictor	Univariate Logistic Regression		
		OR	95% CI OR	*p* *
Sjogren synrome	rs3135932 AA gnotype	2.74	1.048–7.176	0.036 **
	rs3795299 CC genotype	3.87	1.080–13.869	0.029 **
	Anti-SSA antibodies	6.9	2.796–17.045	<0.001 *
Hashimoto thyroiditis	rs9610 GG genotype	3.22	1.106–9.382	0.027
	rs3795299 C allele	4.35	1.165–16.238	0.002
	rs16829204 CC genotype	5	1.074–23.274	0.026
Malar rush	rs10892202 GG genotype	4.37	1.268–15.091	0.013 *
	rs4252270 CC genotype	4.37	1.268–15.091	0.013 *
	rs999788 CC genotype	2.3	1.007–5.251	0.046
	rs2834167 AA genotype	2.61	1.161–5.886	0.019
Photosensitivity	rs2229113GG genotype	2.60	1.024–6.605	0.041 *
Arthritis	rs9610 G allele	3.6	0.947–13.679	0.048
Neurological manifestations	rs1058867 AA genotype	NA	NA	0.011
Hemolytic anemia	rs10892202 C allele	11.591	1.740–77.203	0.018 *
	rs4252270 T allele	11.591	1.740–77.203	0.018 *
	rs3795299 GG genotype	NA	NA	0.014
Lymphopenia	rs3795299 G allele	3.85	0.975–15.168	0.042
Hematological disorders	rs3795299 G allele	4.53	1.302–15.785	0.018 *
Alopecia	rs9610 A allele	2.9	1.110–7.585	0.026 *
AntiSm antibodies	rs16829204 T allele	1.64	1.014–2.640	0.040

* Remain significant after adjustment for age and gender. ** Additionally adjusted for anti-SSA antibody presence. NA—not applicable.

## Data Availability

Data are available upon reasonable request. Please contact the corresponding author. They are not publicly available to ensure the strict confidentiality of the patients’ data.
